# Short Notes on Diseases of the Palate

**Published:** 1889-04

**Authors:** V. A. Latham


### Short Notes on Diseases of the Palate.

V. A. LATHAM, B&C., F.R.M.S., (LOND).

The palate, owing to its structural continuity with the alveoli and gums in front, and its connection posteriorly with the tongue and pharynx by means of the pillars of the fauces, is liable to implication by the extension of disease from these parts; whilst, on the other hand, morbid processes originating in the palate may spread to the neighboring portions of the mouth, fauces, or pharynx. It is necessary to recollect that the palate with the posterior pillars of the fauces constitute a septum between the nasal and buccal passages, which by the contraction of the palato-pharyngei becomes complete during the act of deglutition, and then cuts off all communication between the two cavities; and that its structural and functional integrity is essential for perfect articulation, suction, and deglutition. Hence, the chief symptoms induced by lesions of the part are usually dependent upon impairment of these physiological acts. The diseases of the palate are —(1) congenital malformations, as cleft palate, (2) inflammation, (3) ulcers, (4) necrosis, (5) tumors.

I. *Inflammation* may consist of a simple phlegmon of the soft parts; or in the case of the hard palate may involve the periosteum and bone.

(a) *Phlegmonons inflammation* is rare, except as a result of extension from the tonsils and other adjacent parts. In the velum palati it is accompanied by considerable swelling, but this sign is absent in the hard palate owing to the intimate adherence of the mucous membrane to the bone. It may terminate in abscess.

(B) *Syphilitic Angina* which coincides in period with the early cutaneous manifestations is usually most marked in the palate. It appears as a diffuse redness, with more or less swelling of the mucous membrane of the fauces, and is often of so little intensity that it may be entirely unnoticed by the patient, but occasionally it assumes a parenchymatous form and may lead to abscess-formation or even to sloughing of the soft parts. It may also become chronic lasting two or three months.

(T) *Osteo-periostitis* may arise from traumatic causes, extension of alveolar inflammation, or from syphilitic or tubercular cachexia. The disease is indicated by a local swelling, at first hard, then fluctuating; and there is more or less pain (slight in the syphilitic form) in mastication and deglutition. Should abscess supervene the bone usually perishes and its bared surface may be felt through the opening of the discharge. The separation of the sequestrum may leave a temporary or permanent fistulous communication between the mouth and nose.

2. *Ulcers*—The principal forms of ulceration of the palate are of syphilitic, tubercular, or epitheliomatous origin. Simple ulcers are rare except as a result of local injury and present no special features of interest. Syphilitic ulcers may be superficial or deep, as in the rest of the bucco-pharyngeal tract. The former is common in the early secondary stages of the disease; the latter belongs to the tertiary period and results from the breaking down of a gummatous infiltration.

The deep ulcer appears late, from five to eight years after the primary lesion and is generally single. If situated upon the hard palate it is nearly always central and involves the bone; if the soft palate be the point of attack great destruction and deformity may result, sometimes complicated with adhesions to the posterior wall of the pharynx. In rare instances the tertiary ulcer may spread to the base of the cranium. The sore has the usual character. Tubercular ulcers are most frequently developed in childhood. They usually reach the palate by extension from the posterior pillars of the fauces and are associated with tubercular disease elsewhere. The lesion generally makes its first appearance as small tubercles, which project slightly above the surface; these break down into a number of erosions which soon fuse into an irregular ulcer of little depth with festooned borders and a yellowish base covered with pale granulations. The sore thus formed tends to travel slowly in a serpiginous course, but seldom leads to serious destruction. Epitheliomatous ulcers are excessively rare as primary lesions of the palate, but may extend to the part from the gum or tonsil (q. v.)

3. *Tumours.* In addition to inflammatory, tubercular, and gummatous swellings the palate is subject to a considerable number of true tumour-growths which may be briefly given as follows: cysts, mucous, dermoid, fibroma, enchondroma, and osteoma, myxoma, lipoma, and adenoma.

The glandular tumor is by far the most common of the neoplasms. It is often found upon the hard palate, generally implicating its lower wall on one or the other side, and may reach the size of an egg or even attain larger dimensions. It is soft and elastic to the touch, painless and insensitive and causes no symptoms beyond inconvenience by its encroaching upon the buccal cavity. Structurally it is the type of the salivary glands, is invested by a capsule, and is united to surrounding parts by a somewhat loose connected tissue. The glandular elements of the soft palate may undergo hypertrophic changes by which the thickness of the velum becomes greatly augmented, a condition perhaps analogous to the enlargement of the lip in strumous children. Erectile tumors are almost confined to the hard palate, but the velum may be implicated by a nevous extending from the inner surface of the cheek. Papillomata in form, small, usually pedunculated growths, and not very infrequent are mostly attached to the posterior border of soft palate and to the uvula. Sarcoma, rare tumours, is usually the round-celled variety, but in one instance I believe were found to consist of fusiform and myeloid cells. Epithelioma rarely developed primarily in the palate, may involve the part in association with the tonsils and nasal fossæ, etc. The thyrhoyoid and carotid glands become affected in an early period of the disease.

*Treatment.*—This in regard to inflammatory and ulcerative affections of the palate should be conducted on the same principles as in other parts of the mouth (as in stomatitis) such as good food, pure air, detergent waters (chlor. potash, borax, etc.) ice and such tonics, perchloride of iron in large doses (ʒi—ʒiii three or four times a day). Of the tumors the rarer kinds as lipomata cysts, etc., by using the knife. Adenomata most easily enucleated with the finger nails or curette. Erectile tumors are best treated by means of coagulent injections or galvano-puncture. Papillomata cut off with the thermo-cautery scissors. Sarcomata, if not too extensive, excised. Epithelioma and carcinoma are seldom met with in a condition susceptible of any but alleviative treatment; but if small, may be removed or their destruction may be tried with the galvano-cautery.

The chief danger attached to operation of palatal tumors is the ingress of blood into the trachea, an accident very liable when the patient is under chloroform. Cocaine painted upon the surface, or injected into the substance of the part will allow of a painless removal of the growth while the patient is conscious and in some degree able to guard the air passages. If it should be necessary to induce general anæsthesia or asphyxia, the operation may be performed while the head hangs down over the end of the operating table, so that the blood passes into the walls, or plug the back of the fauces after a preliminary tracheotomy, For further information see Garreston, Erichson, Heath, Holmes Bryant, and works on surgery.

